# Current Status and Hospital-Level Differences in Care and Outcomes of Patients With Acute Non-ST-Segment Elevation Myocardial Infarction in China: Insights From China Acute Myocardial Infarction Registry

**DOI:** 10.3389/fcvm.2021.800222

**Published:** 2022-01-17

**Authors:** Qinghao Zhao, Haiyan Xu, Xuan Zhang, Yunqing Ye, Qiuting Dong, Rui Fu, Hui Sun, Xinxin Yan, Xiaojin Gao, Jingang Yang, Yang Wang, Yuejin Yang

**Affiliations:** ^1^Department of Cardiology, Fuwai Hospital, National Center for Cardiovascular Diseases, Chinese Academy of Medical Sciences and Peking Union Medical College, Beijing, China; ^2^Medical Research and Biometrics Center, Fuwai Hospital, National Center for Cardiovascular Diseases, Chinese Academy of Medical Sciences and Peking Union Medical College, Beijing, China

**Keywords:** non-ST elevated acute myocardial infarction, in-hospital care, invasive strategies, medications, healthcare quality, outcome

## Abstract

**Background:**

With the growing burden of non-ST-elevation myocardial infarction (NSTEMI), developing countries face great challenges in providing equitable treatment nationwide. However, little is known about hospital-level disparities in the quality of NSTEMI care in China. We aimed to investigate the variations in NSTEMI care and patient outcomes across the three hospital levels (province-, prefecture- and county-level, with decreasing scale) in China.

**Methods:**

Data were derived from the China Acute Myocardial Infarction Registry on patients with NSTEMI consecutively registered between January 2013 and November 2016 from 31 provinces and municipalities throughout mainland China. Patients were categorized according to the hospital level they were admitted to. Multilevel generalized mixed models were fitted to examine the relationship between the hospital level and in-hospital mortality risk.

**Results:**

In total, 8,054 patients with NSTEMI were included (province-level: 1,698 patients; prefecture-level: 5,240 patients; county-level: 1,116 patients). Patients in the prefecture- and county-level hospitals were older, more likely to be female, and presented worse cardiac function than those in the province-level hospitals (*P* <0.05). Compared with the province-level hospitals, the rate of invasive strategies was significantly lower in the prefecture- and county-level hospitals (65.3, 43.3, and 15.4%, respectively, *P* <0.001). Invasive strategies were performed within the guideline-recommended timeframe in 25.4, 9.7, and 1.7% of very-high-risk patients, and 16.4, 7.4, and 2.4% of high-risk patients in province-, prefecture- and county-level hospitals, respectively (both *P* <0.001). The use of dual antiplatelet therapy in the county-level hospitals (87.2%) remained inadequate compared to the province- (94.5%, *P* <0.001) and prefecture-level hospitals (94.5%, *P* <0.001). There was an incremental trend of in-hospital mortality from province- to prefecture- to county-level hospitals (3.0, 4.4, and 6.9%, respectively, *P*-trend <0.001). After stepwise adjustment for patient characteristics, presentation, hospital facilities and in-hospital treatments, the hospital-level gap in mortality risk gradually narrowed and lost statistical significance in the fully adjusted model [Odds ratio: province-level vs. prefecture-level: 1.23 (0.73–2.05), *P* = 0.441; province-level vs. county-level: 1.61 (0.80–3.26), *P* = 0.182; *P*-trend = 0.246].

**Conclusions:**

There were significant variations in NSTEMI presentation and treatment patterns across the three hospital levels in China, which may largely explain the hospital-level disparity in in-hospital mortality. Quality improvement initiatives are warranted, especially among lower-level hospitals.

## Introduction

Non-ST-elevation myocardial infarction (NSTEMI) is a major cause of death worldwide, and its incidence continues to rise in both developing and developed countries (1–4). The application of evidence-based treatment is an effective approach to reduce mortality from NSTEMI (5, 6). Over the past decades, there has been a declining trend in the in-hospital mortality of NSTEMI in some countries, primarily due to enhanced compliance with guideline-recommended management (2, 3, 7).

Despite the rapidly growing burden, quality improvement initiatives for NSTEMI care remain inadequate compared with the comprehensive efforts to improve the care for patients with ST-elevation myocardial infarction (STEMI). Recent data from Europe have reported significant hospital-level variations in the quality of NSTEMI care, with widespread suboptimal use of guideline-recommended management (7, 8). Developing countries, particularly China, may face greater challenges in providing optimal and equitable treatment across the nation due to the vast geographic area and uneven economic development (9). However, there is limited information concerning the hospital-level disparities in NSTEMI care provision, treatment patterns, and patient outcomes in China. Filling this gap will provide valuable insight for policymakers, hospital administrators, and clinical practitioners in China and other countries at a similar stage of development.

**Figure 1:**
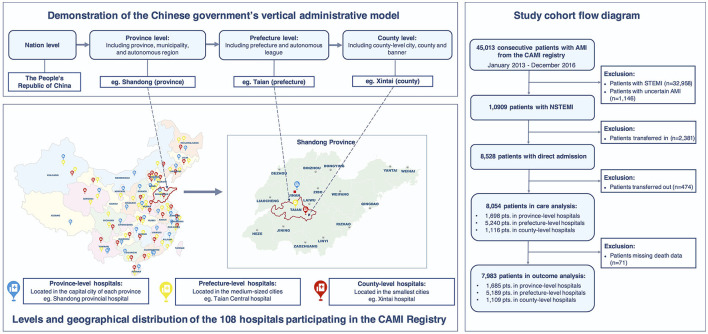
Demonstration of the levels and geographical distribution of the hospitals participating in the CAMI Registry and study cohort flow diagram. The Chinese public hospital system follows a three-level structure based on the Chinese government's vertical administrative model: province-level hospitals (located in the capital cities of each province, Blue marker), prefecture-level hospitals (located in the medium-sized cities, Yellow marker), and county-level hospitals (located in the smallest cities, Red marker), in the order of decreasing scale and level. The CAMI Registry included 108 hospitals covering the three hospital levels from 31 provinces and municipalities throughout Mainland China.

The public hospital system in China follows a traditional structure based on the Chinese government's vertical administrative model, consisting of three levels (province, prefecture, and county) in the order of decreasing scale and level (Figure 1). Province-level hospitals are usually university-affiliated academic hospitals in the capital cities of each province; prefecture-level hospitals are located in the medium-sized cities; county-level hospitals service the smallest cities usually adjacent to rural areas. In general, the bed numbers, availability of hospital facilities and staffing ratios of specialists to generalists decrease as the hospital level decreases. Thus, hospital-level comparisons of NSTEMI management are appropriate and would provide a good picture of the hierarchical performance of the healthcare system in China. Based on the China AMI (CAMI) Registry, a nationwide multicenter prospective observational study for AMI care across the three hospital levels, this study aimed to investigate the hospital-level variations in NSTEMI care and patient outcomes in China.

## Methods

### Overview of the CAMI Registry

The study design of the CAMI Registry has been described previously (10). In brief, the CAMI registry is a prospective, nationwide, multicenter, observational study for AMI care in China. 108 hospitals from 31 provinces and municipalities throughout Mainland China participated since January 2013 (Supplementary Table 1). The participating sites were instructed to enroll consecutive patients with a primary diagnosis of AMI, with a standardized set of variables and standard definitions. The registry included 31 province-level hospitals, 45 prefecture-level hospitals, and 32 county-level hospitals with a wide geographical distribution, covering both urban and rural areas (Figure 1). These hospitals are the largest or the central hospitals in their administrative regions; therefore, a representative sample of local healthcare quality is ensured. In the province-, prefecture- and county-level hospitals, the median bed number in the cardiology units was 122, 83, and 47, respectively; the availability rate of the cardiac-coronary care unit (CCU) was 100, 96, and 78%, respectively; the availability rate of the catheterization laboratory was 100, 93, and 44%, respectively.

The CAMI Registry consecutively enrolled patients with a primary diagnosis of AMI, including STEMI and NSTEMI, who were admitted within 7 days of symptoms onset. The final diagnosis of NSTEMI must meet the 3rd Universal Definition for Myocardial Infarction (11). Type 4a and 5 AMIs were not eligible. Data collection and quality control are detailed in Supplementary Methods. This project was approved by the Institution Review Board Central Committee at Fuwai Hospital, National Center for Cardiovascular Diseases of China. Written informed consent was obtained from all the eligible patients.

### Study Population

In total, 10,909 patients with a confirmed diagnosis of NSTEMI were consecutively enrolled from January 2013 to December 2016. To avoid referral bias and obtain true pre-hospital information, we excluded 2,381 patients who were transferred in and 474 patients who were transferred out. Thus, 8,054 patients with direct admission were included in the core cohort for the care analysis. Moreover, after further exclusion of 71 patients with missing data on death, 7,983 patients were included in the analysis of in-hospital outcomes (Figure 1).

### Variables in Care and Outcomes

The key variable in NSTEMI care was the use of an invasive strategy, i.e., coronary angiography with subsequent revascularization if necessary. We analyzed the procedure timing based on the risk criteria mandating invasive strategy proposed by the current ESC and AHA/ACC guidelines (5, 6). Patients were classified into the very-high-risk category (recommending an immediate invasive strategy <2 h from admission) if they presented with hemodynamic instability or cardiogenic shock, recurrent or ongoing chest pain refractory to medical treatment, life-threatening arrhythmias or cardiac arrest, mechanical complications, acute heart failure, or recurrent dynamic ST-T wave changes. Other patients were classified in the high-risk category (recommending an early invasive strategy <24 h from admission) due to the changes in cardiac biomarkers compatible with AMI. Revascularization procedures, intra-aortic balloon pump (IABP), and medications during hospitalization were also assessed. The primary outcome was in-hospital mortality. Secondary outcomes included in-hospital heart failure, cardiogenic shock, severe arrhythmias, re-infarction, cerebrovascular accident or stroke, and non-intracranial hemorrhage bleeding (detailed definitions in Supplementary Methods).

### Statistical Analyses

Patient characteristics, presentation, treatment, and in-hospital outcomes were compared across the three hospital levels. The normality of continuous data was tested using the Shapiro-Wilk test. Continuous variables with a non-normal distribution were expressed as the median and interquartile range (IQR) and compared using the non-parametric Kruskal-Wallis *H*-test. Categorical variables were presented as percentages with 95% confidence intervals (CIs) and compared using the Chi-square test. The Cochran-Armitage test was performed to examine trends in the crude rates of outcomes across the three hospital levels. To account for within-hospital clustering, we fitted multilevel generalized mixed models with hospitals as a random effect to evaluate the association between the hospital level and in-hospital mortality, adjusting for confounders as a fixed effect. To comprehensively adjust for potential confounders and avoid model over-fitting, the variables included in the adjustment models were carefully chosen. We initially examined all baseline variables by univariate analyses (Supplementary Table 8). Baseline variables with *P* < 0.05 in the univariate analyses or with clinical relevance reported in the previous studies were selected (12–14). The following variables were stepwise incorporated to fit the model 1-6: Model 1 adjusting for patient characteristics (age, sex, hypertension, diabetes, prior myocardial infarction, prior heart failure); Model 2 adjusting for Model 1 plus medical contact (onset-to-arrival time, means of transport); Model 3 adjusting for Model 2 plus clinical status at admission (anterior-wall infarction, systolic blood pressure, heart rate, cardiogenic shock, heart failure, cardiac arrest, Killip class); Model 4 adjusting for Model 3 plus hospital facilities (coronary care unit availability, coronary catheter lab availability); Model 5 adjusting for Model 4 plus the use of medications (aspirin, P_2_Y_12_-receptor inhibitors, statin, β-blocker, angiotensin-converting enzyme inhibitor/angiotensin receptor blocker) and IABP; Model 6 adjusting for Model 5 plus the use of invasive strategies. We reported the odds ratios (ORs) with 95% CIs and tested the linear trend across the three hospital levels in each model. In-hospital mortality was also compared across the three hospital levels in the subgroups categorized by the guideline-recommended risk criteria and the use of invasive strategies. All variables were missing <10% and the multivariate analyses were based on the complete data. We also performed multiple imputations using a Markov chain Monte Carlo algorithm for the missing values and performed multivariate analyses based on the imputed data for sensitivity analyses (15). Statistical significance was set at two-tail *P* < 0.05. All analyses were performed using R 4.0.2 (R Foundation for Statistical Computing, Vienna, Austria).

## Results

### Patient Characteristics and Presentation

Of the 8,054 NSTEMI patients with direct admission, 1,698, 5,240, and 1,116 patients were admitted to province-, prefecture-, and county-level hospitals, respectively. As shown in Table 1 and Supplementary Table 3, compared with patients in the province-level hospitals, patients in the prefecture- and county-level hospitals were older, and more likely to be female and have a history of stroke, but less likely to be obese, or have smoking habits, diabetes, dyslipidemia, prior revascularization procedures, and peripheral artery diseases (all *P* < 0.05). Patients in the prefecture- and county-level hospitals were less likely to present ≤ 24 h after symptoms onset, with less frequency to use ambulance transportations compared to those in the province-level hospitals (both *P* < 0.001). At admission, more patients in the prefecture- and county-level hospitals presented with heart failure, cardiogenic shock, and Killip class III/IV compared with those in the province-level hospitals (all *P* < 0.05). The proportion of very-high-risk patients did not differ significantly across the three hospital levels (*P* = 0.285).

**Table 1:** Baseline characteristics of patients with NSTEMI among the three hospital levels.

**Characteristics**	**Total (*n* = 8,054)**	**Province-level (*n* = 1,698)**	**Prefecture-level (*n* = 5,240)**	**County-level (*n* = 1,116)**	***P*-value**
Age, years	66 (58-75)	64 (55-73)	66 (58-75)	69 (61-77)	<0.001
≥75 years, *n*/*N*(%)	2,108/7,957 (26.5)	354/1,692 (20.9)	1,377/5,157 (26.7)	377/1,108 (34.0)	<0.001
Male, *n*/*N*(%)	5,399/8,054 (67.0)	1,256/1,698 (74.0)	3,523/5,240 (67.2)	620/1,116 (55.6)	<0.001
**Risk factors and medical history**					
BMI, kg/m^2^	24.09 (22.15-25.95)	24.22 (22.49-26.17)	24.06 (22.21-25.90)	23.44 (21.35-25.71)	<0.001
≥25 kg/m^2^, *n*/*N*(%)	2782/7,828 (35.5)	659/1,623 (40.6)	1774/5,108 (34.7)	349/1,097 (31.8)	<0.001
Current smoker, *n*/*N*(%)	2642/7,763 (34.0)	700/1,653 (42.3)	1652/5,015 (32.9)	290/1,095 (26.5)	<0.001
Hypertension, *n*/*N*(%)	4663/7,811 (59.7)	1026/1,658 (61.9)	3001/5,055 (59.4)	636/1,098 (57.9)	0.083
Diabetes history, *n*/*N*(%)	1906/7,799 (24.4)	457/1,652 (27.7)	1229/5,049 (24.3)	220/1,098 (20.0)	<0.001
Known dyslipidemia, *n*/*N*(%)	609/7,803 (7.8)	244/1,656 (14.7)	309/5,049 (6.1)	56/1,098 (5.1)	<0.001
Prior MI, *n*/*N*(%)	1019/7,788 (13.1)	204/1,650 (12.4)	697/5,040 (13.8)	118/1,098 (10.7)	0.013
Prior PCI, *n*/*N*(%)	470/7,777 (6.0)	136/1,646 (8.3)	303/5,036 (6.0)	31/1,095 (2.8)	<0.001
Prior CABG, *n*/*N*(%)	75/7,788 (1.0)	29/1,650 (1.8)	44/5,041 (0.9)	2/1,097 (0.2)	<0.001
Prior heart failure, *n*/*N*(%)	515/7,792 (6.6)	90/1,649 (5.5)	310/5,046 (6.1)	115/1,097 (10.5)	<0.001
Prior stroke, *n*/*N*(%)	903/7,787 (11.6)	153/1,647 (9.3)	615/5,044 (12.2)	135/1,096 (12.3)	0.003
PAD, *n*/*N*(%)	102/7,785 (1.3)	35/1,645 (2.1)	59/5,044 (1.2)	8/1,096 (0.7)	0.003
**Presentation**					
Means of transport, *n*/*N*(%)					<0.001
Self/family	6,798/7,811 (87.0)	1,373/1,659 (82.8)	4,468/5,053 (88.4)	957/1,099 (87.1)	
Ambulance	826/7,811 (10.6)	245/1,659 (14.8)	471/5,053 (9.3)	110/1,099 (10.0)	
In-hospital	187/7,811 (2.4)	41/1,659 (2.5)	114/5,053 (2.3)	32/1,099 (2.9)	
Onset-to-arrival time, *n*/*N*(%)					<0.001
<3 h	1,721/8,048 (21.4)	341/1,694 (20.1)	1,121/5,238 (21.4)	259/1,116 (23.2)	
3-12 h	2,563/8,048 (31.8)	594/1,694 (35.1)	1,664/5,238 (31.8)	305/1,116 (27.3)	
12-24 h	939/8,048 (11.7)	240/1,694 (14.2)	577/5,238 (11.0)	122/1,116 (10.9)	
1-7 days	2,586/8,048 (32.1)	485/1,694 (28.6)	1,726/5,238 (33.0)	375/1,116 (33.6)	
Uncertain	239/8,048 (3.0)	34/1,694 (2.0)	150/5,238 (2.9)	55/1,116 (4.9)	
Anterior MI, *n*/*N*(%)	2,401/7,607 (31.6)	472/1,608 (29.4)	1,533/4,904 (31.3)	396/1,095 (36.2)	0.001
Heart failure on admission, *n*/*N*(%)	1,662/7,801 (21.3)	252/1,658 (15.2)	1,044/5,045 (20.7)	366/1,098 (33.3)	<0.001
Cardiogenic shock on admission, *n*/*N*(%)	186/7,798 (2.4)	21/1,658 (1.3)	119/5,043 (2.4)	46/1,097 (4.2)	<0.001
Cardiac arrest, *n*/*N*(%)	54/7,760 (0.7)	8/1,645 (0.5)	35/5,026 (0.7)	11/1,089 (1.0)	0.282
Systolic blood pressure, mmHg	133 (120-150)	130 (119-150)	133 (119-150)	140 (120-160)	<0.001
Heart rate, bpm	78 (68-90)	76 (67-86)	78 (68-90)	80 (70-100)	<0.001
Killip class III/IV, *n*/*N*(%)	979/7,774 (12.6)	129/1,645 (7.8)	670/5,033 (13.3)	180/1,096 (16.4)	<0.001
GRACE risk score	154 (131-177)	149 (126-169)	156 (132-179)	158 (136-185)	<0.001
>140, *n*/*N*(%)	4,888/7,587 (64.4)	929/1,615 (57.5)	3,204/4,904 (65.3)	755/1,068 (70.7)	<0.001
**Guideline-recommended risk criteria mandating invasive strategy**					
Very-high risk, *n*/*N*(%)	2,858/7,838 (36.5)	602/1,665 (36.2)	1,831/5,072 (36.1)	425/1,101 (38.6)	0.285

*BMI, body mass index; CABG, coronary artery bypass graft; MI, myocardial infarction; GRACE, Global Registry of Acute Coronary Events; PAD, peripheral artery disease; PCI, percutaneous coronary intervention; NSTEMI, Non-ST-segment elevation myocardial infarction*.

### Invasive Strategies and Medication Use

**Figure 2:**
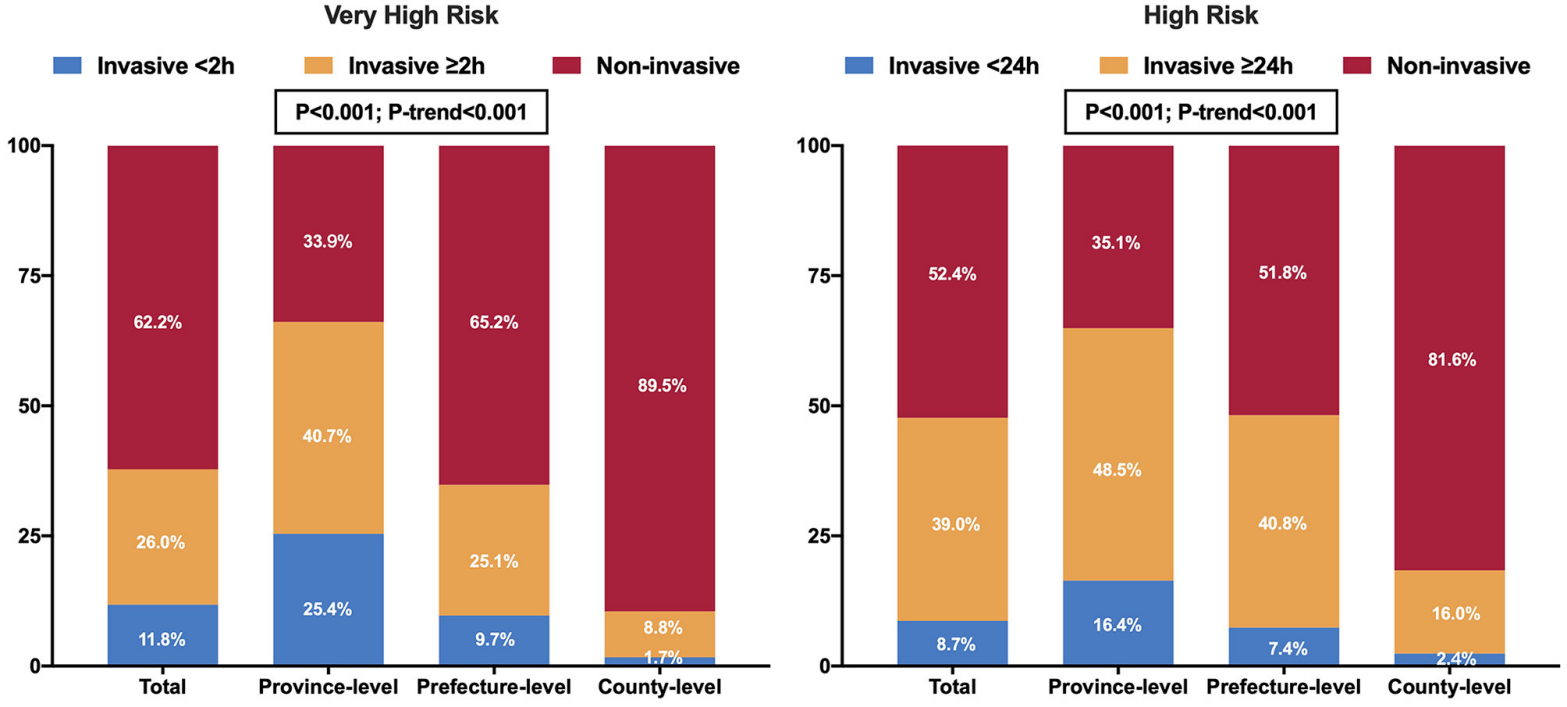
Utilization and timing of invasive strategies in patients with NSTEMI according to the guideline-recommended risk criteria among the three hospital levels in China.

**Figure 3:**
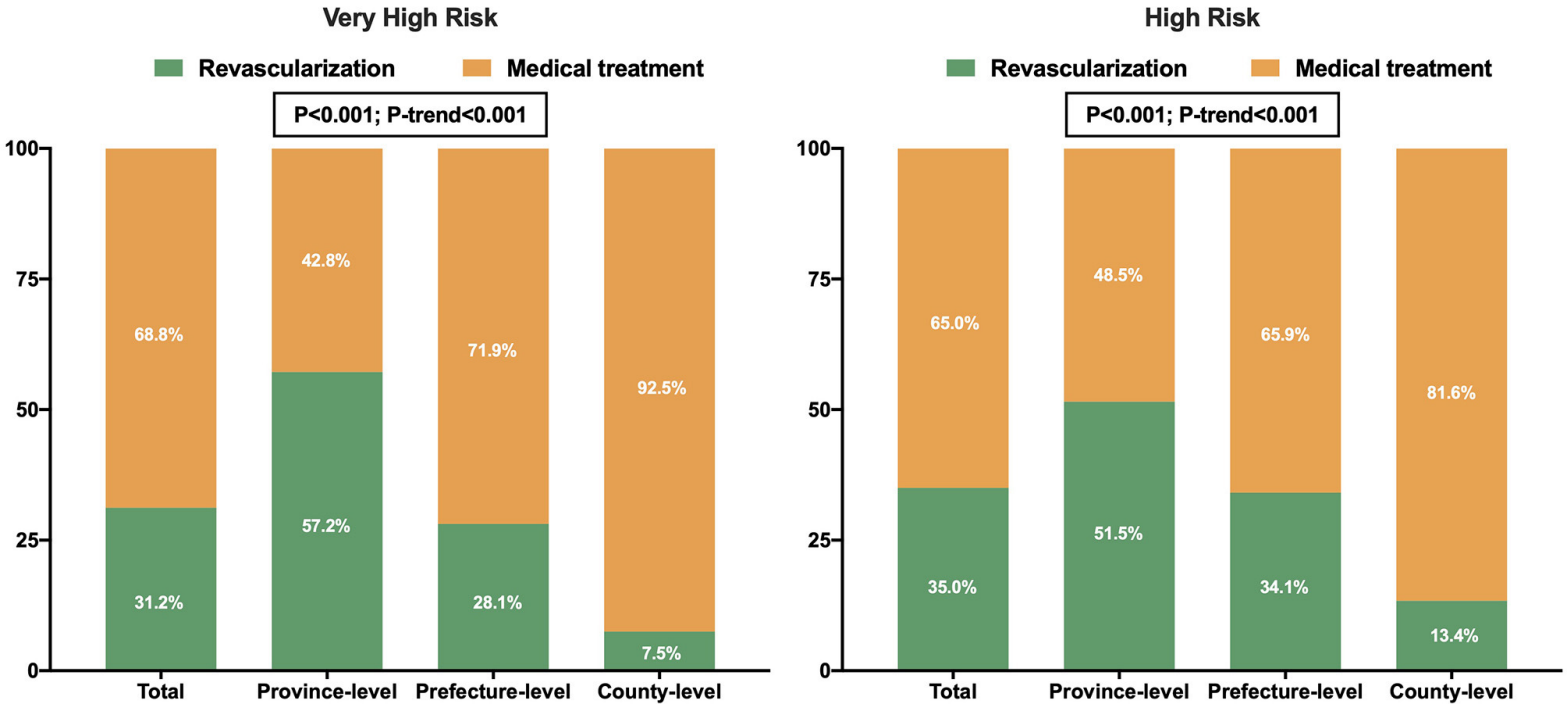
Revascularization rates in patients with NSTEMI according to the guideline-recommended risk criteria among the three hospital levels in China.

Compared to the province-level hospitals, the rate of invasive strategies (coronary angiography with subsequent revascularization if necessary) was significantly lower in the prefecture-level and county-level hospitals [65.3% (63.0–67.6%), 43.3% (42.0–44.7%) and 15.4% (13.2–17.5%), respectively, *P* < 0.001] (Table 2). Among the province-, prefecture- and county-level hospitals, 25.4% (21.9-28.9%), 9.7% (8.4-11.1%), and 1.7% (0.4-2.9%) of very-high-risk patients, and 16.4% (14.2-18.6%), 7.4% (6.5–8.3%), and 2.4% (1.2–3.5%) of high-risk patients were treated with invasive strategies within the guideline-recommended timeframe, respectively (<2 h for very-high-risk; <24 h for high-risk) (both *P* < 0.001 and *P*-trend < 0.001, Figure 2) (5, 6). During hospitalization, 57.2% (53.2–61.2%), 28.1% (26.0–30.2%), and 7.5% (5.0–10.1%) of very-high-risk patients, and 51.5% (48.5–54.5%), 34.1% (32.4–35.7%), and 13.4% (10.8–16.0%) of high-risk patients underwent revascularization in the province-, prefecture- and county-level hospitals, respectively (*P* < 0.001 and *P*-trend < 0.001, Figure 3). IABP was used more frequently in the province-level hospitals than in the prefecture- and county-level hospitals (*P* < 0.001). The overall use of dual antiplatelet therapy was 93.5%. However, this rate was much lower in the county-level hospitals [87.2% (85.3–89.2%)] than in the province-level [94.5% (93.4–95.6%), *P* < 0.001] and prefecture-level hospitals [94.5% (93.9–95.2%), *P* < 0.001]. Statin, β-blocker, and angiotensin-converting enzyme inhibitor/angiotensin receptor blocker (ACEI/ARB) were prescribed in 96.9, 71.7, and 63.9% of overall patients, respectively. Similarly, the use of these drugs was much lower in the county-level hospitals (93.7, 69.4, and 65.5%, respectively) compared with the province-level hospitals (97.5, 77.3, and 67.3%, respectively. All *P* < 0.05) (Table 2).

**Table 2:** Treatments for patients with NSTEMI among the three hospital levels.

**Treatments**	**Total (*n* = 8,054)**	**Province-level (*n* = 1,698)**	**Prefecture-level (*n* = 5,240)**	**County-level (*n* = 1,116)**	***P*-value**
**Procedure**					
Invasive strategy (angiography and subsequent revascularization), *n*/*N*(%)	3,436/7,797 (44.1)	1,083/1,658 (65.3)	2,184/5,039 (43.3)	169/1,100 (15.4)	<0.001
PCI, *n*/*N*(%)	2,510/7,702 (32.6)	840/1,623 (51.8)	1,550/4,989 (31.1)	120/1,090 (11.0)	<0.001
Stent implantation, *n*/*N*(%)	2,101/2,506 (83.8)	738/837 (88.2)	1,259/1,549 (81.3)	104/120 (86.7)	<0.001
DES, *n*/*N*(%)	1,862/1,974 (94.3)	649/684 (94.9)	1,119/1,194 (93.7)	94/96 (97.9)	0.184
PTCA, *n*/*N*(%)	391/2,506 (15.6)	93/837 (11.1)	282/1,549 (18.2)	16/120 (13.3)	<0.001
CABG, *n*/*N*(%)	110/7,706 (1.4)	48/1,610 (3.0)	60/5,005 (1.2)	2/1,091 (0.2)	<0.001
IABP use, *n*/*N*(%)	112/7,693 (1.5)	50/1,638 (3.1)	59/4,981 (1.2)	3/1,074 (0.3)	<0.001
**Medication during hospitalization**					
Aspirin, *n*/*N*(%)	7,425/7,767 (95.6)	1,572/1,648 (95.4)	4,820/5,028 (95.9)	1,033/1,091 (94.7)	0.216
P_2_Y_12_-receptor inhibitor, *n*/*N*(%)	7,494/7,775 (96.4)	1,623/1,655 (98.1)	4,896/5,030 (97.3)	975/1,090 (89.4)	<0.001
Dual antiplatelet therapy, *n*/*N*(%)	7,258/7,762 (93.5)	1,559/1,649 (94.5)	4,748/5,023 (94.5)	951/1,090 (87.2)	<0.001
Statin, *n*/*N*(%)	7,203/7,433 (96.9)	1,544/1,584 (97.5)	4,685/4,809 (97.4)	974/1,040 (93.7)	<0.001
β-blocker, *n*/*N*(%)	5,531/7,710 (71.7)	1,265/1,636 (77.3)	3,512/4,987 (70.4)	754/1,087 (69.4)	<0.001
ACEI/ARB, *n*/*N*(%)	4,926/7,709 (63.9)	1,104/1,640 (67.3)	3,110/4,982 (62.4)	712/1,087 (65.5)	0.001
Heparin/Fondaparinux, *n*/*N*(%)	7,103/7,736 (91.8)	1,431/1,637 (87.4)	4,688/5,011 (93.6)	984/1,088 (90.4)	<0.001
GP IIb/IIIa inhibitor, *n*/*N*(%)	1,309/7,576 (17.3)	424/1,614 (26.3)	753/4,890 (15.4)	132/1,072 (12.3)	<0.001
Traditional Chinese Medicine, *n*/*N*(%)	1,347/7,663 (17.6)	231/1,621 (14.3)	932/4,963 (18.8)	184/1,079 (17.1)	<0.001
**Length of stay, days**	10 (7–14)	8 (6–12)	11 (7–14)	9 (6–13)	<0.001
**Medication at discharge**					
Aspirin, *n*/*N*(%)	7,167/7,659 (93.6)	1,565/1,627 (96.2)	4,629/4,962 (93.3)	973/1,070 (90.9)	<0.001
P_2_Y_12_-receptor inhibitor, *n*/*N*(%)	7,105/7,638 (93.0)	1,599/1,626 (98.3)	4,608/4,942 (93.2)	898/1,070 (83.9)	<0.001
Dual antiplatelet therapy, *n*/*N*(%)	6,886/7,629 (90.3)	1,549/1,623 (95.4)	4,462/4,937 (90.4)	875/1,069 (81.9)	<0.001
Statin, *n*/*N*(%)	6,959/7,318 (95.1)	1,532/1,559 (98.3)	4,502/4,739 (95.0)	925/1,020 (90.7)	<0.001
β-blocker, *n*/*N*(%)	5,145/7,615 (67.6)	1,184/1,615 (73.3)	3,277/4,932 (66.4)	684/1,068 (64.0)	<0.001
ACEI/ARB, *n*/*N*(%)	4,606/7,608 (60.5)	1,001/1,618 (61.9)	2,929/4,922 (59.5)	676/1,068 (63.3)	0.033

*ACEI, angiotensin-converting enzyme inhibitor; ARB, angiotensin receptor blocker; CABG, coronary artery bypass graft; DES, drug-elute stent; IABP, intra-aortic balloon pump; GP, glycoprotein; PCI, percutaneous coronary intervention; NSTEMI, Non-ST-segment elevation myocardial infarction*.

### In-Hospital Outcomes

**Figure 4:**
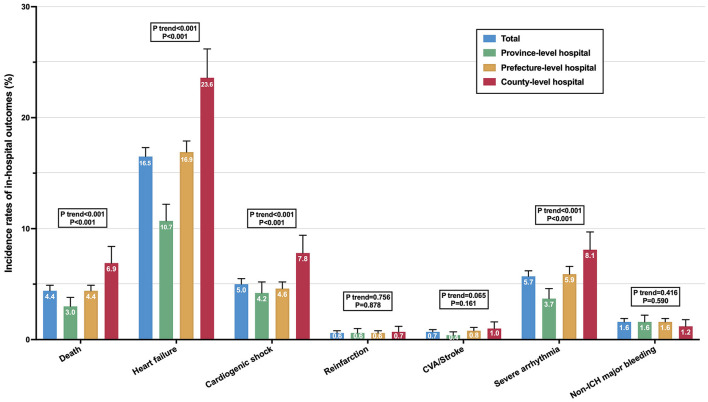
Incidence of in-hospital outcomes in patients with NSTEMI among the three hospital levels in China. The error bars indicate the 95% confidence interval of the sampling errors.

There was an incremental trend of in-hospital mortality from the province- to prefecture- to county-level hospitals [3.0% (2.2–3.8%), 4.4% (3.8–4.9%), 6.9% (5.4–8.4%), *P*-trend < 0.001, Figure 4]. Compared to the province-level hospitals, the odds of in-hospital mortality were 1.62 (95% CI: 1.03–2.56, *P* = 0.038) and 2.50 (95% CI: 1.49–4.17, *P* < 0.001) times higher for the prefecture- and county-level hospitals, respectively. This disparity persisted in the subset of very-high-risk patients (*P* = 0.001) and high-risk patients (*P* = 0.033), but was not significant in the subset of patients undergoing invasive strategies [0.7% (0.2–1.3%), 1.2% (0.7–1.6%), 1.2% (0–2.8%), *P* = 0.447] (Supplementary Table 6). There were similar increasing trends in the incidence of heart failure, cardiogenic shock, and severe arrhythmia across the three hospital levels (all *P* < 0.001 and *P*-trend < 0.001, Figure 4).

**Figure 5:**
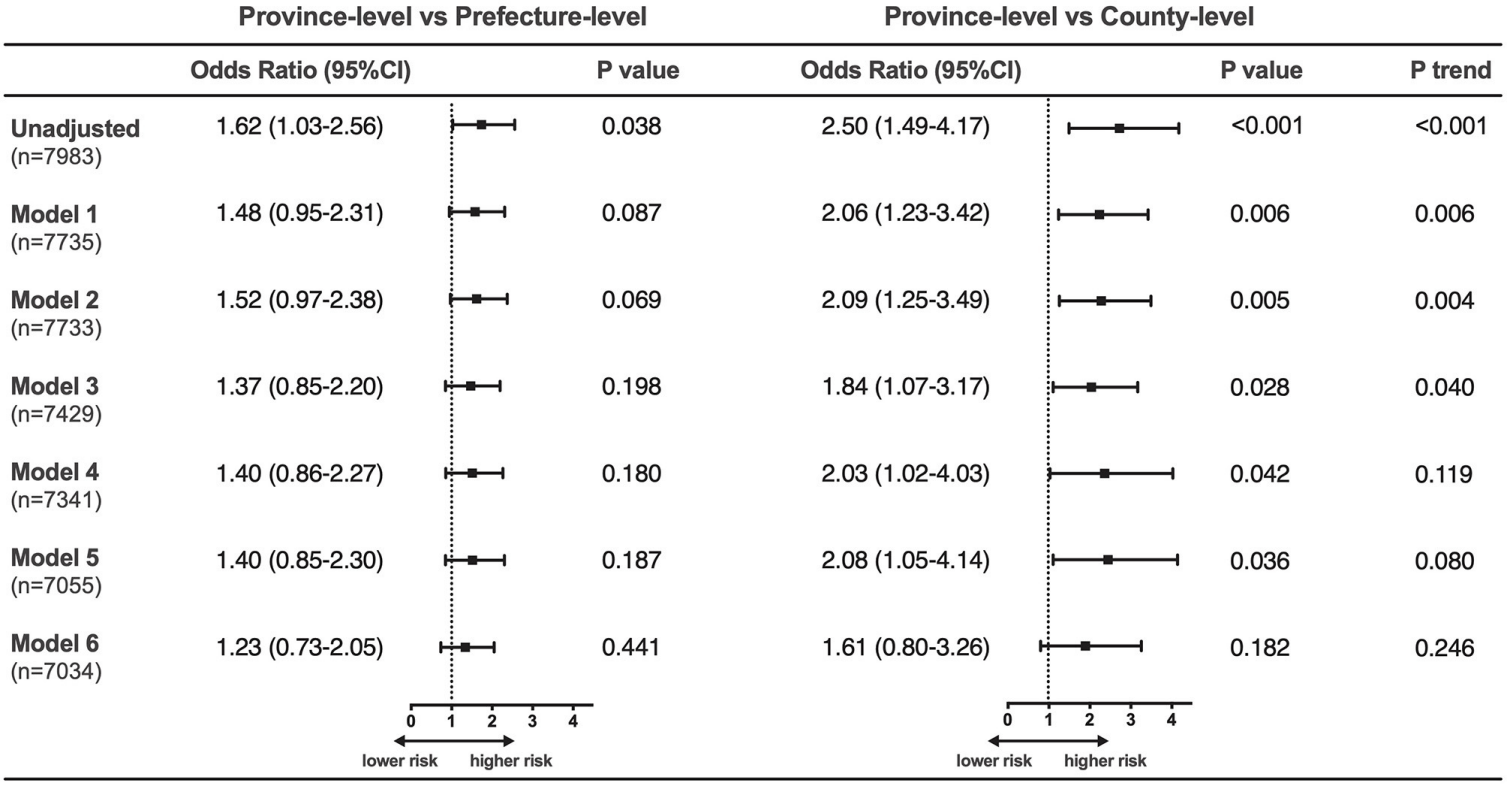
Adjusted in-hospital mortality risk in patients with NSTEMI among the three hospital levels. Model 1 adjusting for patient characteristics (age, sex, hypertension, diabetes, prior myocardial infarction, prior heart failure); Model 2 adjusting for Model 1 plus medical contact (onset-to-arrival time, means of transport); Model 3 adjusting for Model 2 plus clinical status at admission (anterior-wall infarction, systolic blood pressure, heart rate, cardiogenic shock, heart failure, cardiac arrest, Killip class); Model 4 adjusting for Model 3 plus hospital facilities (coronary care unit availability, coronary catheter lab availability); Model 5 adjusting for Model 4 plus the use of medications (aspirin, P_2_Y_12_-receptor inhibitors, statin, β-blocker, angiotensin-converting enzyme inhibitor/angiotensin receptor blocker) and intra-aortic balloon pump; Model 6 adjusting for Model 5 plus the use of invasive strategies.

As Figure 5 shows, after adjustment for patient characteristics and presentation (Model 1–3), there was no significant difference in mortality risk between the province- and prefecture-level hospitals [OR (95% CI): 1.37 (0.85–2.20), *P* = 0.198], but the remarkable disparity between the province- and county-level hospitals persisted [OR (95% CI): 1.84 (1.07–3.17), *P* = 0.028]. After additional adjustment for hospital facilities and the use of medications and IABP (Model 4–5), county-level hospitals were still significantly associated with a higher mortality risk compared with the province-level hospitals [OR (95% CI): 2.08 (1.05–4.14), *P* = 0.036]. However, after adding invasive strategies to the model (Model 6), the gap in mortality risk between the province- and county-level hospitals remarkably narrowed, resulting in a loss of statistical significance [OR (95% CI): 1.61 (0.80–3.26), *P* = 0.182; *P*-trend = 0.246]. Similar results were observed in the sensitivity analysis based on the multiple imputation data (Supplementary Table 9). Furthermore, the use of invasive strategies was identified as the strongest protective factor for mortality of NSTEMI, as attested by its largest Wald Chi-square statistics in the fully adjusted model [OR (95% CI): 0.20 (0.13–0.31), *P* < 0.001, Wald Chi-square = 51.961, Supplementary Table 10].

## Discussion

This study is the first national report on the hospital-level differences in NSTEMI care and patient outcomes in China. The key findings are as follows: First, invasive strategies were underused and markedly delayed in the current management of NSTEMI, especially in lower-level hospitals. Second, the overall use of dual antiplatelet therapy was high, but more improvements in county-level hospitals are desirable. Third, there was an incremental trend of in-hospital mortality with decreasing hospital levels. The hospital-level disparity in mortality could be largely explained by the wide variations in patient presentation and treatment patterns between hospitals, particularly in terms of available invasive strategies. These findings shed light on the challenges that China currently faces, and could not only serve as a basis for improving healthcare quality in China but hold valuable insights for other developing countries.

### Use of Invasive Strategies

The invasive strategies have a central role in NSTEMI care (5, 6). However, the overall rate of invasive strategies in China was low (44.1%), which was markedly lower than the rate in Canada (60.2%), Denmark (63.3%), the United States (79.6%), and South Korea (91.3%) (16–18). Although the province-level hospitals showed a comparable rate of 65.3%, the prominently low rate at 15.4% in the county-level hospitals pulled the average down. There may be several reasons for the limited use of invasive strategies in lower-level hospitals. First, PCI capability varied across the three hospital levels. In our study, the catheterization laboratory was available in 44% of the county-level hospitals compared to 100% of the province-level hospitals. The lack of PCI facilities and interventional specialists directly limits the number of interventional procedures. Second, physicians in lower-level hospitals tended to adopt a more conservative strategy for patients with complex and critical conditions. We observed an inverse relationship between the revascularization rates and patient risk stratification in the prefecture- and county-level hospitals, with a similar risk-treatment paradox reported in previous studies (16, 19, 20). This paradox may be due to concerns about procedure-related complications and inadequate clinical expertise (21). Third, some subjective factors of patients, such as affordability concerns, may have also influenced the decision for or against invasive strategies, especially in lower-level hospitals (9).

Current guidelines strongly endorse the use of an early invasive strategy within 24 h of admission in patients with NSTEMI to reduce ischemic complications and length of in-hospital stay (5, 6). In particular, the invasive strategy should be performed within 2 h if the patient meets the very-high-risk criteria (5). Our study observed substantial in-hospital delays, with only 11.8% of very-high-risk patients and 8.7% of high-risk patients meeting the time targets. This rate is similar to the recent findings of a national registry in the United Kingdom (16.4%) (22), suggesting that delayed application of invasive strategies is a common issue in NSTEMI management. There may be a common time delay in identifying patients with NSTEMI due to their frequent presentation of atypical symptoms and lack of definite electrocardiographic changes. Time delays may also result from the decision-making process for invasive strategies because many NSTEMI patients have type-2 AMI for whom evidence-based treatment is still lacking (22, 23). Moreover, some time-consuming processes, including patient consent provision (patient factor) and preparations for the PCI procedure (hospital factor), may further exacerbate in-hospital delays.

### Use of Medications

Dual antiplatelet therapy, consisting of aspirin and P_2_Y_12_-receptor inhibitor, is the cornerstone of the medical management of AMI (5, 6). The China-PEACE study described a remarkable improvement in the use of evidence-based antiplatelet therapy for patients with AMI in China, with aspirin use increasing from 86.5 to 90.0% and clopidogrel use increasing from 45.7 to 79.8%, from 2006 to 2011 (24, 25). Our data collected between 2013 and 2016 showed that the use of aspirin and P_2_Y_12_-receptor inhibitor in patients with NSTEMI reached 95.6 and 96.4%, respectively. These results are comparable to the rates in developed healthcare systems (18, 22). However, the use of dual antiplatelet therapy in the county-level hospitals (87.2%) remained inadequate compared to the province- and prefecture-level hospitals (both 94.5%). The overall use of other guideline-recommended medications, including statin, β-blocker, and ACEI/ARB, was similar to that reported by the registries in Italy, Switzerland, the United States, and South Korea (18, 26, 27). However, when categorized by hospital levels, the use of statin, β-blocker and ACEI/ARB was much lower in the county-level hospitals compared with the province-level hospitals (all *P* < 0.05). Since few county-level hospitals are tertiary hospitals staffed with cardiovascular specialists, the lack of practitioners with condition-specific expertise may explain the underuse of evidence-based medical therapies in county-level hospitals (24, 25).

### Hospital-Level Disparity in In-Hospital Mortality

Notably, there was a significant disparity in the in-hospital mortality across the three hospital levels, with the highest mortality rate (6.9%) in the county-level hospitals while the lowest rate (3.0%) in the province-level hospitals. This disparity in mortality can only be partially explained by patient-level variations, as the remarkable differences in mortality risk between province- and county-level hospitals persisted, albeit attenuated, after adjusting for patient characteristics and presentation. Of greater importance, factors related to in-hospital care, including hospital facilities, as well as the use of medications, IABP, and invasive strategies, may be more critical determinants of in-hospital mortality. As these factors were stepwise incorporated into the model, the gap in mortality risk between hospitals gradually narrowed. However, it was not until the inclusion of invasive strategies into the model that the disparity in mortality risk between province- and county-level hospitals lost statistical significance. In the fully adjusted model, invasive strategies also had the greatest weight on in-hospital mortality (Supplementary Table 10). Moreover, in the subset of patients who underwent invasive strategies, the mortality rate was remarkably low, ranging from 0.7 to 1.2%, with no significant variations across the three hospital levels. These evidences suggest the crucial role of invasive strategies in determining mortality associated with NSTEMI. Thus, in the contemporary management of NSTEMI, addressing the inequalities in the application of invasive strategies and enhancing the rate of invasive strategies in lower-level hospitals are critical to narrow the hospital-level disparities in mortality.

### Implications for Future Improvement in NSTEMI Care

As mentioned above, for future improvement in NSTEMI care in China, it is of prime importance to reduce the hospital-level inequalities in applying invasive strategies. To achieve this, more investments in the construction and staffing of catheterization laboratories are necessary to provide basic PCI facilities, thereby promoting the use of invasive strategies in lower-level hospitals. Developing regional medical combination networks with optimized processes of timely transferal of patients to PCI-capable hospitals is also a very cost-efficient approach to increase the access to PCI (28, 29). Moreover, greater emphasis should be placed on the intensive training and technical support for the interventional cardiologists in lower-level hospitals, for improving clinical practice and adapting evidence-based therapies. Beginning in 2015, China has been undertaking a major healthcare reform to build a tiered healthcare delivery system, aiming to strengthen the infrastructure in lower-level hospitals, and more importantly, developing medical alliances (30). In this model, the leading hospitals in each alliance not only serve as a centralized location for training programs and fast-track referrals, but also share responsibilities, resources, management, and economic interests with their alliance members (30). This model may help address the gaps in medical resource allocation in China and provide valuable insights for other countries at a similar point of development.

### Study Limitations

This study has certain limitations. First, as not all hospitals participated in this national registry, we could not collect all cases of NSTEMI in China. However, to objectively reflect the quality of NSTEMI care in the Chinese public medical system, our study was uniquely designed to include the hospitals across the three levels rather than the binary comparisons between urban-rural or tertiary-secondary hospitals. Therefore, our comparisons were more consistent with the medical practice pattern and administrative model in China, and can well reflect the hierarchical performance of the Chinese public medical system. Second, as an observational study, the possibility of residual measured and unmeasured confounders may be present. However, we used multilevel mixed models to account for within-hospital clustering and adjust for comprehensive variables relating to multiple facets of presentation and care to minimize bias. Third, the CAMI Registry is a hospital-based registry study that enrolled the patients admitted to hospitals but did not include outpatients. Therefore, we could only estimate the in-hospital mortality of NSTEMI but could not assess the out-of-hospital mortality.

## Conclusion

In this large nationwide analysis of hospital-level differences in NSTEMI care and patient outcomes in China, we found that patient presentation and treatment patterns varied widely across the three hospital levels, which may largely explain the hospital-level disparities in the in-hospital mortality rates. Invasive strategies played a key role in determining mortality associated with NSTEMI. Thus, it is crucial to reduce the hospital-level inequalities in applying invasive strategies and to increase the rate of invasive strategies in patients with NSTEMI. In this scenario, national initiatives and investments in quality improvement, with a particular focus on lower-level hospitals, are warranted for the delivery of optimal and equitable care for patients with NSTEMI. Our findings provide valuable insights for policymakers and medical professionals in China and other developing countries, informing future strategies for healthcare quality improvement and medical resource allocation.

## Data Availability Statement

The raw data supporting the conclusions of this article will be made available by the authors, without undue reservation.

## Ethics Statement

The studies involving human participants were reviewed and approved by Institution Review Board Central Committee at Fuwai Hospital, National Center for Cardiovascular Diseases of China. The patients/participants provided their written informed consent to participate in this study.

## Author Contributions

YYa and HX conceived the study. QZ and HX developed the study methodology. QZ edited the initial draft of the manuscript. XZ, QD, RF, HS, XY, XG, JY, and YW were involved in the study implement, data collection, and data audit. YYa, HX, and YYe critically revised the manuscript for important intellectual content. All authors contributed to the article and approved the submitted version.

## Funding

This work was supported by the Chinese Academy of Medical Sciences Innovation Fund for Medical Sciences [Grant no. CIFMS2016-I2M-1-009] and National Twelfth Five-year Science and Technology Support Projects by Ministry of Science and Technology of China [Grant no. 2011BAI11B02].

## Conflict of Interest

The authors declare that the research was conducted in the absence of any commercial or financial relationships that could be construed as a potential conflict of interest.

## Publisher's Note

All claims expressed in this article are solely those of the authors and do not necessarily represent those of their affiliated organizations, or those of the publisher, the editors and the reviewers. Any product that may be evaluated in this article, or claim that may be made by its manufacturer, is not guaranteed or endorsed by the publisher.

## Abbreviations

NSTEMI: Non-ST-elevation myocardial infarction
STEMI: ST-elevation myocardial infarction
AMI: acute myocardial infarction
CCU: cardiac-coronary care unit
IABP: intra-aortic balloon pump
PCI: percutaneous coronary intervention
CABG: coronary artery bypass grafting.
