# Supplementary material for: Current Status and Hospital-Level Differences in Care and Outcomes of Patients With Acute Non-ST-Segment Elevation Myocardial Infarction in China: Insights From China Acute Myocardial Infarction Registry
Source: Front Cardiovasc Med. 2022 Jan 17;8:800222. doi: 10.3389/fcvm.2021.800222 (PMC8801489; doi:10.3389/fcvm.2021.800222)
Supplement: Supplementary file 1 [file Data_Sheet_1.PDF]

SUPPLEMENTARY MATERIAL

Zhao et al. Current Status and Hospital-level Differences in Care and Outcomes of Patients with Acute Non-ST-segment Elevation Myocardial Infarction in China: Insights from China Acute Myocardial Infarction Registry

Content:

Supplementary Methods

Data collection and quality control of the CAMI Registry study **(P3)**

Definitions of in-hospital clinical events **(P3-5)**

Supplementary Tables

Supplementary Table 1. List of the hospitals participating in the CAMI Registry **(P6-8)**

Supplementary Table 2. Baseline characteristics with 95% confidence intervals of patients with NSTEMI among the three hospital levels in China. **(P9-10)**

Supplementary Table 3. Multi-comparisons of baseline characteristics of patients with NSTEMI among the three hospital levels in China. **(P11-12)**

Supplementary Table 4. Invasive treatments and medication use with 95% confidence intervals in patients with NSTEMI among the three hospital levels in China. **(P13)**

Supplementary Table 5. Multi-comparisons of the invasive treatments and medication use in patients with NSTEMI among the three hospital levels in China. **(P14)**

Supplementary Table 6. In-hospital mortality in patients with NTSEMI categorized by the guideline-recommended risk criteria and the use of invasive strategies among the three hospital levels in China. **(P15)**

Supplementary Table 7. Multi-comparisons of the in-hospital mortality across the three hospital levels in subsets categorized by the guideline-recommended risk criteria and the use of invasive strategies **(P16)**

Supplementary Table 8. Univariable analysis of predictive factors of in-hospital mortality risk in patients with NSTEMI **(P17-18)**

Supplementary Table 9. Adjusted in-hospital mortality risk in patients with NSTEMI across the three hospital levels based on the multiple imputation data. **(P19)**

Supplementary Table 10. Factors associated with the in-hospital mortality in patients with NTSEMI based on the complete data. **(P20-21)**

Supplementary Table 11. Factors associated with the in-hospital mortality in patients with NTSEMI based on the multiple imputation data. **(P22-23)**

Supplementary Methods

Data collection and quality control of the CAMI Registry study

Comprehensive data concerning patient demographics, risk factors, medical history, pre-hospital medical contact, presentation, status at admission, vital signs, invasive management, medications, and clinical outcomes were collected, using a standardized set of variables with predefined definitions and systematic data entry and transmission procedures. Data were submitted through a secure, web-based electronic data capture system (http://www.CAMIRegistry.org). Enrollment, data collection, and follow-up were all performed by trained physicians, cardiologists, or cardiovascular fellows at each participating site in a real-time manner to ensure data accuracy and reliability. Senior cardiologists were responsible for the data quality control. Periodic database checks were performed and queries for illogical, invalid or missing data elements were sent to participating hospitals to review and revise. Hospital sites received random on-site audit for the consecutiveness of enrollment and accuracy of diagnosis based on medical records.

Definitions of In-hospital clinical events

In-hospital death: Patient died during the hospitalization.

Heart Failure: Indicate if there is physician documentation or report of either new onset or acute reoccurrence of the following heart failure symptoms during the hospitalization, including unusual dyspnea on light exertion, recurrent dyspnea occurring in the supine position, fluid retention; or the description of rales, jugular venous distension, pulmonary edema on physical exam, or pulmonary edema on chest x-ray presumed to be cardiac dysfunction. A low ejection fraction without clinical evidence of heart failure does not qualify as heart failure.

Cardiogenic Shock: Indicate if the patient had a new onset or acute recurrence of cardiogenic shock during the hospitalization. Cardiogenic shock is defined as a sustained (>30 minutes) episode of systolic blood pressure <90 mm Hg, and/or cardiac index <2.2 L/min/m^2 determined to be secondary to cardiac dysfunction, and/or the requirement for parenteral inotropic or vasopressor agents or mechanical support (e.g., IABP, extracorporeal circulation, ventricular assist devices) to maintain blood pressure and cardiac index above those specified levels. Transient episodes of hypotension reversed with IV fluid or atropine do not constitute cardiogenic shock. The hemodynamic compromise (with or without extraordinary supportive therapy) must persist for at least 30 minutes.

Arrhythmia: Indicate if the patient had a new episode or acute recurrence of the following arrhythmia during the hospitalization, atrial fibrillation/flutter**,** supraventricular tachycardia requiring treatment (supraventricular tachycardia that requires cardioversion, drug therapy, or is sustained for greater than 1 minute)**,** ventricular tachycardia or ventricular fibrillation**,** sinus pause or bradycardia**,** second- or third-degree atrioventricular block.

Re-infarction: Indicate if there are clinical signs and symptoms of a new infarction or repeat infarction occurring during the hospitalization. Re-infarction is diagnosed if the value of the second cardiac marker sample obtained 3-6 hours later increased by 20% compared to the immediate measurement when the suspected clinical signs or symptoms occurred. This value should also exceed the 99th percentile upper reference limit. Re-infarction is considered when ST elevation >0.1 mV reoccurs in a patient having a lesser degree of ST elevation or new pathological Q waves, in at least two contiguous leads, particularly when associated with ischemic symptoms for 20 min or longer.

Cerebrovascular accident/Stroke: Indicate if the patient experienced a stroke or cerebrovascular accident (CVA) during the hospitalization. A CVA/Stroke is defined as loss of neurological function caused by a cerebrovascular event with residual symptoms at least 24 hours after onset or leading to death, including (1) Hemorrhagic: A stroke with documentation on imaging (e.g., CT scan or MRI of hemorrhage in the cerebral parenchyma, or a subdural or subarachnoid hemorrhage). Evidence of hemorrhagic stroke obtained from lumbar puncture, neurosurgery, or autopsy can also confirm the diagnosis; (2) Ischemic: A focal neurological deficit that results from a thrombus or embolus (and not due to hemorrhage) that appears and is still partially evident for more than 24 hours; (3) Ischemic with hemorrhagic conversion; (4) Unknown: if the type of stroke could not be determined by imaging or other means.

Non-intracranial hemorrhage bleedings: Indicate if there was a suspected or confirmed bleeding event observed and documented in the medical record that was associated with any of the following: (1). Hemoglobin drop of >=3 g/dL; (2). Transfusion of whole blood or packed red blood cells; (3). Procedural intervention/surgery at the bleeding site to reverse/stop or correct the bleeding (such as surgical closures/exploration of the arteriotomy site, balloon angioplasty to seal an arterial tear, endoscopy with cautery of a gastrointestinal bleed)； (4). Medical attention required to stop bleeding.

Supplementary Table 1. List of the hospitals participating in the CAMI Registry

Hospital	Province/Municipality	City	PI
Fuwai Hospital	Beijing	Beijing	Yuan Wu
Beijing Friendship Hospital	Beijing	Beijing	Hongwei Li
Beijing Tongren Hospital	Beijing	Beijing	Changlin Lu
Beijing Daxing Hospital	Beijing	Daxing	Shujun Cao
Beijing Mentougou Hospital	Beijing	Mentougou	Dezhao Wang
Beijing Pinggu Hospital	Beijing	Pinggu	Guanglin Wei
Beijing Yanqing Hospital	Beijing	Yanqing	Jianbing Wang
Shanghai Jiaotong University Ruijin Hospital	Shanghai	Shanghai	Ruiyan Zhang
Shanghai 10th Hospital	Shanghai	Shanghai	Yawei Xu
Shanghai Fengxian Hospital	Shanghai	Fengxian	Zengyong Qiao
Tianjin Medical School General Hospital	Tianjin	Tianjin	Zheng Wan
Tianjin Baodi Hospital	Tianjin	Baodi	Yanjun Cao
Chongqing Medical School 2st Hospital	Chongqing	Chongqing	Yaohui Yin
Haerbin Medical School 1st Affiliated Hospital	Heilongjiang	Harbin	Weiming Li
Qiqihaer 1st Hospital	Heilongjiang	Qiqihar	Shuqing Wang
Tailai Hospital	Heilongjiang	Tailai	Gang Ma
Shuihua 1st Hospital	Heilongjiang	Shuihua	Yongchen Cai
Jilin University 1st Hospital	Jilin	Changchun	Yang Zheng
Tonghua Central Hospital	Jilin	Tonghua	Xuxia Zhang
Huinan County Hospital	Jilin	Huinan	Hongyan Guo
Shenyang Northern Hospital	Liaoning	Shenyang	Xiaozeng Wang
Fushun Central Hospital	Liaoning	Fushun	Ling Sun
Xiuyan County Hospital	Liaoning	Xiuyan	Jianhua Wu
Neimonggu Medical College 1st Affiliated Hospital	Neimenggu	Hohhot	Fengying Chen
Chifeng Hospital	Neimenggu	Chifeng	Ronghai Man
Aohan Hospital	Neimenggu	Aohan	Yanjie Li
Hebei Medcial School 2rd Affiliated Hospital	Hebei	Shijiazhuang	Xianghua Fu
Qinhuangdao 1st Hospital	Hebei	Qinhuangdao	Qingshen Wang
Qinhuangdao 2rd Hospital	Hebei	Changli	Liying Zhang
North-China Oil-administration General Hospital	Hebei	Renqiu	Xiaoli Gao
Changzhou Hospital	Hebei	Changzhou	Yali Hu
Hengshui Hardison Hospital	Hebei	Hengshui	Qun Zheng
Shanxi Cardiovascular Hospital	Shanxi	Taiyuan	Bao Li
Changzhi Hospital	Shanxi	Changzhi	Yuping zhang
Tunliu Hospital	Shanxi	Tunliu	Yaohong Dong
Henan Provincial Hospital	Henan	Zhengzhou	Chuanyu Gao
Linzhou Hospital	Henan	Linzhou	Zhoushun Qin
Changyuan Hospital	Henan	Changyuan	Guorui Hou
Xinxiang Central Hospital	Henan	Xinxiang	Lingling Liu
Yanjin Hospital	Henan	Yanjin	Shifeng Ren
Ye County hospital	Henan	Ye County	Dezhou wang

Hospital	Province/Municipality	City	PI
Pindingshan 2rd Hospital	Henan	Pindingshan	Xianting Luan
Anyang Prefecture Hospital	Henan	Anyang	Hui Liu
Puyang People's Hospital	Henan	Puyang	Liping Ma
Xihua Hospital	Henan	Xihua	Chuntong Wang
Xi'an Jiaotong University 1st Hospital	Shannxi	Xi'an	Zuyi Yuan
Weinan Central Hospital	Shannxi	Weinan	Junnong Li
Jiuquan Hospital	Gansu	Jiuquan	Yaofeng Yuan
Jinta Hospital	Gansu	Jinta	Huide Liu
Ningxia Medical College General Hospital	Ningxia	Yinchuan	Shaobinjia
Wuzhong Hospital	Ningxia	Wuzhong	Xianghong Luo
Qinghai University Affiliated Hospital	Qinghai	Xining	Yin Liu
Qinhai Cardiovascular Hospital	Qinghai	XiOg	Piirfa Liu
Xining 1st Hospital	Qinghai	Xining	Xianning Zhao
Hainan Prefectural Hospital of Qinghai	Qinghai	Gonghe	Bao Ma
Xinjiang Medical College 1st Affiliated Hospital	Xinjiang	Urumcm	YitongMa
Changji Hospital	Xinjiang	Changji	Mao Wang
Fukang Hospital	Xinjiang	Fukang	Shiming Gao
Urumchi Friendship Hospital	Xinjiang	Urumchi	Hang Lu
Shandong Provincial Hospital	Shandong	Jinan	Lianqun Cui
Taian Central Hospital	Shandong	Taian	Huanyi Zhang
Xintai Hospital	Shandong	Xintai	Hongyan Zhang
Nanjing University Gulou Hospital	Jiangsu	Nanjin	Biao Xu
Jiangsu North Hospital	Jiangsu	Yangzhou	Shenghu He
Xuzhou 1st Central Hospital	Jiangsu	Xuzhou	Qiang Fu
Jiangyan Hospital	Jiangsu	Jiangyan	Shihai Shen
Anhui Provincial Hospital	Anhui	Hefei	Likun Ma
Fuyang Hospital	Anhui	Fuyang	Bin Ning
Taihe Hospital	Anhui	Taihe	Jili Fan
Zhejiang University 2rd Affiliated Hospital	Zhejiang	Hangzhou	Yong Sun
Taizhou Enze medical Center	Zhejiang	Taizhou	Lijiang tang
Taizhou Hospital	Zhejiang	Linhai	Danlei Xu
Fujian Medical College Union Hospital	Fujian	Fuzhou	Lianglong Chen
Xiamen Heart Center	Fujian	Xiamen	Yan Wang
Fuqing Hospital	Fujian	Fuqing	Ping chen
Longyan 1st Hospital	Fujian	Longyan	Kaihong Chen
Wuhan Tongji Hospital	Hubei	Wuhan	Daowen wang
Jinzhou 1st Hospital	Hubei	Jinzhou	Shuixian peng
Tianmen 1st Hospital	Hubei	Tianmen	Shuping Wan
Gong'an Hospital	Hubei	Gongan	Laxi Zhang

Hospital	Province/Municipality	City	PI
Central South University Xiangya 2ndHospital	Hunan	Changsha	Shenhua Zhou
Xiangtan Central Hospital	Hunan	Xiangtan	Jianping Zeng
Xiangxiang Hospital	Hunan	Xiangxiang	Chonglun Zhou
Ya'an Hospital	Sichuan	Ya'an	Haibo zhang
Zigong 1st Hospital	Sichuan	Zigong	Dechao Zhong
Danleng County Hospital	Sichuan	Danleng	Yuquan Xiao
Guangxi Medical College 1st Affiliated Hospital	Guangxi	Nanning	Lang Li
Beihai Hospital	Guangxi	Beihai	Hai Zhu
Hepu Hospital	Guangxi	Hepu	Meisheng Lai
Nanchang Universuty 2ndAffiliated Hospital	Jiangxi	Nanchang	Xiaoshu Cheng
Pingxiang Hospital	Jiangxi	Pingxiang	Junming Ye
Shangli Hospital	Jiangxi	Shangli	Qishou Liu
Guizhou Cardiovascular Hospital	Guizhou	Guiyang	Tianhe Yang
Zhunyi 1st Hospital	Guizhou	Zhunyi	Zhengqiang Yuan
Honghuagang Hospital	Guizhou	Honghuagan	Chengyuan Zhao
Pan County Hospital	Guizhou	Pan	Xianwen Jiang
Guangdong Provincial Hospital	Guangdong	Guangzhou	Jiyan Chen
Guangzhou Traditional Chinese Medical College 1st Affiliated Hospital	Guangdong	Guangzhou	WeiWu
Jiangmen Hospital	Guangdong	Jiangmen	Gaoxing Zhang
Heshan Hospital	Guangdong	Heshan	Haiyuan Mai
Kunming Medical College 1st Affiliated Hospital	Yunnan	Kunming	Tao Guo
Yunnan St. John's Hospital	Yunnan	Kunming	Yi Li
Chuxiong People's Hosptal	Yunnan	Chuxiong	Xiaoming Liu
Yao'an Hospital	Yunnan	Yao'an	Jinlong Xu
Tibet People's Hospital	Tibet	Lahsa	Gesang Luobu
Hainan Provincial Hospital	Hainan	Haikou	Bin Li
Sanya Hospital	Hainan	Sanya	Tiansong Wang
Wenchang Hospital	Hainan	Wenchang	Dong Wang

A total of 108 hospitals from 31 provinces and municipalities participated in the CAMI Registry study

Supplementary Table 2. Baseline characteristics with 95% confidence intervals of patients with NSTEMI among the three hospital levels in China.

Characteristics	Province-level	Prefecture-level	County-level
≥75 years, %	20.9 (19.0-22.9)	26.7 (25.5-27.9)	34.0 (31.2-36.8)
Male, %	74.0 (71.9-76.1)	67.2 (66.0-68.5)	55.6 (52.6-58.5)
**Risk factors and medical history**			
BMI≥25 kg/m^2, %	40.6 (38.2-43.0)	34.7 (33.4-36.0)	31.8 (29.1-34.6)
Current smoker, %	42.3 (40.0-44.7)	32.9 (31.6-34.2)	26.5 (23.9-29.1)
Hypertension, %	61.9 (59.5-64.2)	59.4 (58-60.7)	57.9 (55.0-60.8)
Diabetes history, %	27.7 (25.5-29.8)	24.3 (23.2-25.5)	20.0 (17.7-22.4)
Known dyslipidemia, %	14.7 (13.0-16.4)	6.1 (5.5-6.8)	5.1 (3.8-6.4)
Prior MI, %	12.4 (10.8-14.0)	13.8 (12.9-14.8)	10.7 (8.9-12.6)
Prior PCI, %	8.3 (6.9-9.6)	6.0 (5.4-6.7)	2.8 (1.8-3.8)
Prior CABG, %	1.8 (1.1-2.4)	0.9 (0.6-1.1)	0.2 (0-0.4)
Prior heart failure, %	5.5 (4.4-6.6)	6.1 (5.5-6.8)	10.5 (8.7-12.3)
Prior stroke, %	9.3 (7.9-10.7)	12.2 (11.3-13.1)	12.3 (10.4-14.3)
PAD, %	2.1 (1.4-2.8)	1.2 (0.9-1.5)	0.7 (0.2-1.2)
**Presentation**			
Means of transport, %			
Self/family	82.8 (80.9-84.6)	88.4 (87.5-89.3)	87.1 (85.1-89.1)
Ambulance	14.8 (13.1-16.5)	9.3 (8.5-10.1)	10.0 (8.2-11.8)
In-hospital	2.5 (1.7-3.2)	2.3 (1.8-2.7)	2.9 (1.9-3.9)
Onset-to-arrival time, %			
<3h	20.1 (18.2-22)	21.4 (20.3-22.5)	23.2 (20.7-25.7)
3-12h	35.1 (32.8-37.3)	31.8 (30.5-33)	27.3 (24.7-29.9)
12-24h	14.2 (12.5-15.8)	11.0 (10.2-11.9)	10.9 (9.1-12.8)
1-7 days	28.6 (26.5-30.8)	33.0 (31.7-34.2)	33.6 (30.8-36.4)
Uncertain	2.0 (1.3-2.7)	2.9 (2.4-3.3)	4.9 (3.7-6.2)
Anterior MI, %	29.4 (27.1-31.6)	31.3 (30.0-32.6)	36.2 (33.3-39.0)
Heart failure on admission, %	15.2 (13.5-16.9)	20.7 (19.6-21.8)	33.3 (30.5-36.1)

Cardiogenic shock on admission, %	1.3 (0.7-1.8)	2.4 (1.9-2.8)	4.2 (3.0-5.4)
Cardiac arrest, %	0.5 (0.1-0.8)	0.7 (0.5-0.9)	1.0 (0.4-1.6)
Killip III/IV, %	7.8 (6.5-9.1)	13.3 (12.4-14.3)	16.4 (14.2-18.6)
GRACE risk score >140, %	57.5 (55.1-59.9)	65.3 (64-66.7)	70.7 (68.0-73.4)
Guideline-recommended risk criteria mandating invasive strategy			
Very-high risk, %	36.2 (33.8-38.5)	36.1 (34.8-37.4)	38.6 (35.7-41.5)

Abbreviation: BMI= body mass index; CABG= coronary artery bypass graft; MI= myocardial infarction; GRACE= Global Registry of Acute Coronary Events; PAD= peripheral artery disease; PCI= percutaneous coronary intervention; NSTEMI= Non-ST-segment elevation myocardial infarction;

Supplementary Table 3. Multi-comparisons of baseline characteristics of patients with NSTEMI among the three hospital levels in China.

Characteristics	P value: Province- vs prefecture-level	P value: Province- vs county-level	P value: prefecture- vs county-level
≥75 years	<0.001	<0.001	<0.001
Male	<0.001	<0.001	<0.001
**Risk factors and medical history**			
BMI≥25 kg/m^2	<0.001	<0.001	0.064
Current smoker	<0.001	<0.001	<0.001
Hypertension	0.069	0.038	0.378
Diabetes history	0.007	<0.001	0.002
Known dyslipidemia	<0.001	<0.001	0.187
Prior MI	0.127	0.195	0.005
Prior PCI	0.002	<0.001	<0.001
Prior CABG	0.004	<0.001	0.011
Prior heart failure	0.303	<0.001	<0.001
Prior stroke	0.001	0.012	0.909
PAD	0.006	0.002	0.182
**Presentation**			
Means of transport	<0.001	0.001	0.336
Self/family			
Ambulance			
In-hospital			
Onset-to-arrival time	<0.001	<0.001	0.001
<3h			
3-12h			
12-24h			
1-7 days			
Uncertain			
Anterior MI	0.149	<0.001	0.002

Heart failure on admission	<0.001	<0.001	<0.001
Cardiogenic shock on admission	0.004	<0.001	0.001
Cardiac arrest	0.341	0.111	0.296
Killip III/IV	<0.001	<0.001	0.008
GRACE risk score >140	<0.001	<0.001	0.001
Guideline-recommended risk criteria mandating invasive strategy			
Very-high risk	0.967	0.193	0.119

Abbreviation: BMI= body mass index; CABG= coronary artery bypass graft; MI= myocardial infarction; GRACE= Global Registry of Acute Coronary Events; PAD= peripheral artery disease; PCI= percutaneous coronary intervention; NSTEMI= Non-ST-segment elevation myocardial infarction;

Supplementary Table 4. Invasive treatments and medication use with 95% confidence intervals in patients with NSTEMI among the three hospital levels in China.

**Treatments**	**Province-level**	**Prefecture-level**	**County-level**
**Procedure**			
Invasive strategy (angiography), %	65.3 (63.0-67.6)	43.3 (42.0-44.7)	15.4 (13.2-17.5)
PCI, %	51.8 (49.3-54.2)	31.1 (29.8-32.4)	11.0 (9.1-12.9)
Stent implantation, %	88.2 (86.0-90.4)	81.3 (79.3-83.2)	86.7 (80.5-92.8)
DES, %	94.9 (93.2-96.5)	93.7 (92.3-95.1)	97.9 (95.0-100)
PTCA, %	11.1 (9-13.2)	18.2 (16.3-20.1)	13.3 (7.2-19.5)
CABG, %	3.0 (2.1-3.8)	1.2 (0.9-1.5)	0.2 (0-0.4)
IABP use, %	3.1 (2.2-3.9)	1.2 (0.9-1.5)	0.3 (0-0.6)
**Medication during hospitalization**			
Aspirin, %	95.4 (94.4-96.4)	95.9 (95.3-96.4)	94.7 (93.4-96.0)
P_2Y_{12}-receptor inhibitor, %	98.1 (97.4-98.7)	97.3 (96.9-97.8)	89.4 (87.6-91.3)
Dual antiplatelet therapy, %	94.5 (93.4-95.6)	94.5 (93.9-95.2)	87.2 (85.3-89.2)
Statin, %	97.5 (96.7-98.2)	97.4 (97.0-97.9)	93.7 (92.2-95.1)
β-blocker, %	77.3 (75.3-79.4)	70.4 (69.2-71.7)	69.4 (66.6-72.1)
ACEI/ARB, %	67.3 (65.0-69.6)	62.4 (61.1-63.8)	65.5 (62.7-68.3)
Heparin/Fondaparinux, %	87.4 (85.8-89.0)	93.6 (92.9-94.2)	90.4 (88.7-92.2)
GP IIb/IIIa inhibitor, %	26.3 (24.1-28.4)	15.4 (14.4-16.4)	12.3 (10.3-14.3)
Traditional Chinese Medicine, %	14.3 (12.5-16.0)	18.8 (17.7-19.9)	17.1 (14.8-19.3)

Abbreviation: ACEI= angiotensin-converting enzyme inhibitor; ARB= angiotensin receptor blocker; CABG= coronary artery bypass graft; DES= drug-elute stent; GP= glycoprotein; IABP= intra-aortic balloon pump; PCI= percutaneous coronary intervention; NSTEMI= Non-ST-segment elevation myocardial infarction;

Supplementary Table 5. Multi-comparisons of the invasive treatments and medication use in patients with NSTEMI among the three hospital levels in China.

Treatments	P value: Province- vs prefecture-level	P value: Province- vs county-level	P value: prefecture- vs county-level
**Procedure**			
Coronary angiography	<0.001	<0.001	<0.001
PCI	<0.001	<0.001	<0.001
Stent implantation	<0.001	0.640	0.127
DES	0.297	0.301	0.115
PTCA	<0.001	0.482	0.165
CABG	<0.001	<0.001	0.001
IABP use	<0.001	<0.001	0.004
**Medication during hospitalization**			
Aspirin	0.411	0.405	0.091
P_2Y_{12}-receptor inhibitor	0.089	<0.001	<0.001
Dual antiplatelet therapy	0.970	<0.001	<0.001
Statin	0.907	<0.001	<0.001
β-blocker	<0.001	<0.001	0.490
ACEI/ARB	<0.001	0.325	0.056
Heparin/Fondaparinux	<0.001	0.014	<0.001
GP IIb/IIIa inhibitor	<0.001	<0.001	0.009
Traditional Chinese Medicine	<0.001	0.049	0.182
Length of stay	<0.001	0.258	<0.001

Abbreviation: ACEI= angiotensin-converting enzyme inhibitor; ARB= angiotensin receptor blocker; CABG= coronary artery bypass graft; DES= drug-elute stent; GP= glycoprotein; IABP= intra-aortic balloon pump; PCI= percutaneous coronary intervention; NSTEMI= Non-ST-segment elevation myocardial infarction;

Supplementary Table 6. In-hospital mortality in patients with NTSEMI categorized by the guideline-recommended risk criteria and the use of invasive strategies among the three hospital levels in China.

	**Total (n=7983)**	**Province-level (n=1685)**	**Prefecture-level (n=5189)**	**County-level (n=1109)**	**p value**
Mortality, n/N(%)	355/7983 (4.4)	51/1685 (3.0)	227/5189 (4.4)	77/1109 (6.9)	<0.001
Invasive strategy					
Yes	36/3431 (1.0)	8/1082 (0.7)	26/2181 (1.2)	2/168 (1.2)	0.447
No	319/4340 (7.4)	43/573 (7.5)	201/2840 (7.1)	75/927 (8.1)	0.589
Risk stratification					
Very-high risk	238/2844 (8.4)	33/600 (5.5)	153/1823 (8.4)	52/421 (12.4)	0.001
High risk	117/4959 (2.4)	18/1059 (1.7)	74/3225 (2.3)	25/675 (3.7)	0.033

Abbreviation: NSTEMI= Non-ST-segment elevation myocardial infarction.

Supplementary Table 7. Multi-comparisons of the in-hospital mortality across the three hospital levels in subsets categorized by the guideline-recommended risk criteria and the use of invasive strategies

	P value for Province- vs Prefecture-level	P value for Province- vs County-level	P value for Prefecture- vs County-level
Overall	0.012	<0.001	<0.001
Invasive strategy			
Yes	0.217	0.632	0.998
No	0.719	0.681	0.309
Risk stratification			
Very-high risk	0.017	<0.001	0.014
High risk	0.235	0.010	0.044

Abbreviation: NSTEMI= Non-ST-segment elevation myocardial infarction.

Supplementary Table 8. Univariable analysis of predictive factors of in-hospital mortality risk in patients with NSTEMI

Variables	Odds ratio (95% confidence interval)	P value
**Patients characteristics**		
Age	1.063 (1.052-1.074)	<0.001
Sex	0.597 (0.481-0.740)	<0.001
BMI	0.920 (0.888-0.953)	<0.001
Hypertension	1.335 (1.066-1.672)	0.012
Diabetes	1.527 (1.214-1.919)	<0.001
Known dyslipidemia	0.533 (0.258-1.101)	0.089
Prior myocardial infarction	1.565 (1.187-2.063)	0.001
Prior PCI	0.630 (0.366-1.085)	0.096
Prior CABG	0.873 (0.274-2.786)	0.819
Prior heart failure	3.414 (2.562-4.549)	<0.001
Prior stroke	1.948 (1.485-2.555)	<0.001
Peripheral artery diseases	0.852 (0.312-2.329)	0.755
**Medical contact**		
Onset-to-arrival time ≤24 hours	0.939 (0.748-1.179)	0.590
Ambulance use	0.655 (0.484-0.887)	0.006
**Clinical status at admission**		
Anterior-wall infarction	1.215 (0.969-1.523)	0.091
Heart failure on admission	4.346 (3.502-5.393)	<0.001
Cardiogenic shock on admission	12.639 (9.112-17.532)	<0.001
Cardiac arrest on admission	8.320 (4.542-15.241)	<0.001
Systolic blood pressure on admission	0.976 (0.971-0.980)	<0.001
Heart rate on admission	1.017 (1.012-1.021)	<0.001
Killip class III/IV	5.391 (4.310-6.743)	<0.001
**Hospital facilities**		
Coronary care unit availability	0.484 (0.317-0.740)	0.001
Coronary catheter lab availability	0.540 (0.404-0.721)	<0.001

**Treatment**		
Invasive strategy	0.134 (0.094-0.189)	<0.001
IABP	3.301 (1.896-5.748)	<0.001
Aspirin	0.223 (0.163-0.305)	<0.001
P2Y12-receptor inhibitor	0.225 (0.161-0.316)	<0.001
Statin	0.200 (0.140-0.287)	<0.001
β-blocker	0.310 (0.250-0.385)	<0.001
ACEI/ARB	0.379 (0.305-0.472)	<0.001

Abbreviation: ACEI= angiotensin-converting enzyme inhibitor; ARB= angiotensin receptor blocker; BMI= body mass index; CABG= coronary artery bypass graft; IABP= intra-aortic balloon pump; NSTEMI= Non-ST-segment elevation myocardial infarction; PCI= percutaneous coronary intervention;

Supplementary Table 9. Adjusted in-hospital mortality risk in patients with NSTEMI across the three hospital levels based on the multiple imputation data

Hospital level	Model	Adjusted OR (95%CI)	p value
**Province-level vs Prefecture-level**	Unadjusted	1.62 (1.03-2.56)	0.038
	Model 1: baseline characteristics	1.49 (0.96-2.31)	0.075
	Model 2: plus medical contact	1.53 (0.99-2.38)	0.058
	Model 3: plus status at presentation	1.38 (0.86-2.22)	0.180
	Model 4: plus hospital facility	1.42 (0.87-2.30)	0.160
	Model 5: plus use of medication and IABP	1.42 (0.88-2.30)	0.156
	Model 6: plus use of invasive strategies	1.23 (0.75-2.02)	0.417
**Province-level vs County-level**	Unadjusted	2.50 (1.49-4.17)	<0.001
	Model 1: baseline characteristics	2.08 (1.26-3.43)	0.004
	Model 2: plus medical contact	2.12 (1.28-3.51)	0.003
	Model 3: plus status at presentation	1.87 (1.09-3.22)	0.024
	Model 4: plus hospital facility	2.08 (1.06-4.10)	0.034
	Model 5: plus use of medication and IABP	2.07 (1.06-4.05)	0.033
	Model 6: plus use of invasive strategies	1.64 (0.83-3.26)	0.158
**Prefecture-level vs County-level**	Unadjusted	1.56 (1.08-2.26)	0.017
	Model 1: baseline characteristics	1.42 (0.98-2.06)	0.065
	Model 2: plus medical contact	1.41 (0.97-2.05)	0.069
	Model 3: plus status at presentation	1.38 (0.91-2.11)	0.132
	Model 4: plus hospital facility	1.45 (0.83-2.53)	0.193
	Model 5: plus use of medication and IABP	1.28 (0.88-1.87)	0.199
	Model 6: plus use of invasive strategies	1.34 (0.77-2.33)	0.308

Abbreviation: IABP= intra-aortic balloon pump; NSTEMI= Non-ST-segment elevation myocardial infarction;

Adjusted variates: Model 1 adjusting for patient characteristics (age, sex, hypertension, diabetes, prior myocardial infarction, prior heart failure); Model 2 adjusting for Model 1 plus medical contact (onset-to-arrival time, means of transport); Model 3 adjusting for Model 2 plus clinical status at admission (anterior-wall infarction, systolic blood pressure, heart rate, cardiogenic shock, heart failure, cardiac arrest, Killip class); Model 4 adjusting for Model 3 plus hospital facilities (coronary care unit availability, coronary catheter lab availability); Model 5 adjusting for Model 4 plus the use of medications (aspirin, P2Y12-receptor inhibitor, statin, β-blocker, angiotensin-converting enzyme inhibitor/angiotensin receptor blocker) and intra-aortic balloon pump; Model 6 adjusting for Model 5 plus the use of invasive strategies.

Supplementary Table 10. Factors associated with in-hospital mortality in patients with NSTEMI based on the complete data.

Fixed effects factors	Wald χ2 statistics	Adjusted OR (95%CI)	P value
**Hospital level**	1.782		
Prefecture-level (vs Province-level hospital)		1.61 (0.80-3.26)	0.182
County-level (vs Province-level hospital)		1.23 (0.73-2.05)	0.441
**Patients characteristics**			
Age	23.558	1.03 (1.02-1.05)	<0.001
Sex	0.145	0.95 (0.73-1.23)	0.703
Hypertension	14.352	1.72 (1.30-2.27)	<0.001
Diabetes	5.189	1.39 (1.05-1.84)	0.023
Prior myocardial infarction	0.229	1.09 (0.77-1.53)	0.632
Prior heart failure	0.554	1.16 (0.79-1.71)	0.457
**Medical contact**			
Onset-to-arrival time ≤24 hours	0.004	0.99 (0.76-1.29)	0.947
Ambulance use	1.124	1.23 (0.84-1.79)	0.289
**Clinical status at admission**			
Anterior-wall infarction	1.394	1.18 (0.90-1.54)	0.238
Systolic blood pressure on admission	50.743	0.98 (0.98-0.99)	<0.001
Heart rate on admission	6.586	1.01 (1.00-1.01)	0.010
Cardiogenic shock on admission	5.828	1.85 (1.12-3.04)	0.016
Heart failure on admission	8.718	1.61 (1.17-2.20)	0.003
Cardiac arrest on admission	0.51	1.40 (0.55-3.57)	0.475
Killip class III/IV	9.466	1.66 (1.20-2.30)	0.002
**Hospital facilities**			
Coronary care unit availability	3.761	0.48 (0.23-1.01)	0.053
Coronary catheter lab availability	2.492	1.77 (0.87-3.59)	0.115
**Treatment**			
Invasive strategy	51.961	0.20 (0.13-0.31)	<0.001
IABP	25.054	7.26 (3.34-15.78)	<0.001

Aspirin	7.055	0.55 (0.35-0.85)	0.008
P2Y12-receptor inhibitors	0.405	1.19 (0.70-2.02)	0.524
Statin	4.970	0.57 (0.34-0.93)	0.026
β-blocker	18.050	0.56 (0.43-0.73)	<0.001
ACEI/ARB	18.495	0.54 (0.41-0.72)	<0.001
**Random effects factors**		**Random intercepts (95%CI)**	**P value**
Hospitals		0.29 (0.13-0.63)	0.012

Abbreviation: ACEI= angiotensin-converting enzyme inhibitor; ARB= angiotensin receptor blocker; IABP= intra-aortic balloon pump.

Supplementary Table 11. Factors associated with in-hospital mortality in patients with NSTEMI based on the multiple imputation data.

Fixed effects factors	Wald χ2 statistics	Adjusted OR (95%CI)	P value
**Hospital level**	1.992		
Prefecture-level (vs Province-level hospital)		1.64 (0.83-3.26)	0.158
County-level (vs Province-level hospital)		1.23 (0.75-2.02)	0.417
**Patients characteristics**			
Age	23.220	1.03 (1.02-1.04)	<0.001
Sex	0.499	0.92 (0.72-1.17)	0.480
Hypertension	20.776	1.86 (1.43-2.44)	<0.001
Diabetes	8.429	1.49 (1.14-1.95)	0.004
Prior myocardial infarction	0.503	1.13 (0.81-1.56)	0.478
Prior heart failure	0.844	1.19 (0.82-1.72)	0.358
**Medical contact**			
Onset-to-arrival time ≤24 hours	0.189	1.06 (0.82-1.36)	0.664
Ambulance use	1.172	1.22 (0.85-1.75)	0.279
**Clinical status at admission**			
Anterior-wall infarction	2.409	1.23 (0.95-1.58)	0.121
Systolic blood pressure on admission	56.247	0.98 (0.98-0.99)	<0.001
Heart rate on admission	9.737	1.01 (1.00-1.01)	0.002
Cardiogenic shock on admission	8.572	2.00 (1.26-3.17)	0.003
Heart failure on admission	13.899	1.77 (1.31-2.39)	<0.001
Cardiac arrest on admission	6.821	2.96 (1.31-6.69)	0.009
Killip class III/IV	11.983	1.73 (1.27-2.36)	0.001
**Hospital facilities**			
Coronary care unit availability	3.866	0.48 (0.23-0.99)	0.049
Coronary catheter lab availability	1.254	1.48 (0.75-2.95)	0.263
**Treatment**			
Invasive strategy	53.952	0.21 (0.14-0.31)	<0.001
IABP	30.624	7.85 (3.79-16.30)	<0.001

Aspirin	3.006	0.68 (0.43-1.05)	0.083
P2Y12-receptor inhibitor	4.085	1.69 (1.02-2.82)	0.043
Statin	1.196	0.81 (0.55-1.19)	0.274
β-blocker	16.833	0.58 (0.45-0.75)	<0.001
ACEI/ARB	18.521	0.55 (0.42-0.73)	<0.001
**Random effects factors**		**Random intercepts (95%CI)**	**P value**
Hospitals		0.30 (0.14-0.64)	0.009

Abbreviation: ACEI= angiotensin-converting enzyme inhibitor; ARB= angiotensin receptor blocker; IABP= intra-aortic balloon pump.
